# ERK signaling promotes IKKε expression and oncogenic functions in pancreatic cancer cells in association with TBK1

**DOI:** 10.1016/j.jbc.2025.110535

**Published:** 2025-07-28

**Authors:** Adam Graves, Angana Mukherjee, Runying Yang, Angie Mordant, Thomas Webb, Kirsten Bryant, Laura Herring, Albert S. Baldwin

**Affiliations:** 1 Department of Pharmacology, University of North Carolina at Chapel Hill, Chapel Hill, North Carolina, USA; 2 UNC Lineberger Comprehensive Cancer Center, University of North Carolina at Chapel Hill, Chapel Hill, North Carolina, USA; 3 UNC Michael Hooker Metabolomics and Proteomics Core, University of North Carolina at Chapel Hill, Chapel Hill, North Carolina, USA

**Keywords:** MAPK signaling, ERK, pancreatic cancer, KRAS, IKKε, TBK1

## Abstract

A variety of cancers utilize RAS-regulated signaling pathways to promote oncogenic phenotypes. A widely studied example is pancreatic cancer, where mutant KRAS signaling leads to activation of MEK (mitogen-activated protein kinase/extracellular signal–regulated kinase [ERK])–ERK and downstream signaling, which promotes oncogenic mechanisms, including cell proliferation. Importantly, ERK inhibitors have shown efficacy in some cancer clinical trials. Previously, others have studied the effects of the related kinases TANK-binding kinase 1 (TBK1) and inhibitor of nuclear factor kappa-B kinase epsilon (IKKε) in pancreatic cancer where they have been shown to promote cell survival. Here, we show that RAS–mitogen-activated protein kinase signaling promotes expression of IKKε through control of protein stability and not through control of RNA levels. RNA-Seq analysis indicates that TBK1 and IKKε contribute to the expression of a subset of ERK-regulated genes. Potentially related to the effects on IKKε, proteomic analysis reveals that ERK functions to stabilize a relatively large set of proteins independent of RNA regulation. Knockdown of IKKε and TBK1 individually does not affect growth of MIA PaCa-2 pancreatic cancer cells, but dual knockdown significantly inhibits MIA PaCa-2 growth, which is mediated through cell death. Concurrent silencing or inhibition of both TBK1 and IKKε also reduces tumor sphere growth in MIA PaCa-2 cells, correlating with a loss of stemness pathways found with RNA-Seq. The data suggest the importance of regulation of IKKε by ERK in pancreatic cancer cells and of the combined oncogenic activity of TBK1 and IKKε.

Pancreatic cancer has an extremely low 5-year survival rate because of inefficient early detection and aggressive properties leading to metastasis and therapeutic resistance (1, 2). While traditional pancreatic cancer therapies involve chemoirradiation therapy, newer approaches focus on targeting intrinsic cancer-associated signaling pathways (2). A critical oncogenic driver in most pancreatic cancers is Kirsten's rat sarcoma virus (KRAS), with activating mutations being present in over 90% of cancers (2, 3). KRAS, a small GTPase, drives the activation of several downstream pathways, including the PI3K–Akt, guanine nucleotide dissociation stimulator (RalGDS), and the mitogen-activated protein kinase (MAPK) signaling cascades. The MAPK signaling pathway comprises Raf, MEK1/2 (mitogen-activated protein kinase/extracellular signal–regulated kinase 1/2), and extracellular signal–regulated kinase 1/2 (ERK1/2). ERK1/2 (hereafter referred to as ERK), phosphorylates numerous substrates within the cell, several of which regulate proliferation, invasion, and cell division (3, 4). Success with small-molecule inhibitors targeting ERK have led to an increased interest in its functions and downstream activity as well as understanding its crucial role in the progression of cancer, including pancreatic cancer (5). Recent publications have further defined the extensive role of ERK in pancreatic cancer, including its role as a cell cycle regulator, although specific mechanisms linking ERK with an oncogenic role have yet to be described (6, 7, 8).

The IKK family-related kinases, TANK-binding kinase 1 (TBK1) and inhibitor of nuclear factor kappa-B kinase epsilon (IKKε), have been studied in innate immunity as mediators of the intracellular response to pathogenic invasion (9). Downstream of Toll-like receptors and DNA sensors, such as cyclic GMP-AMP synthase/stimulator of interferon genes, TBK1, and IKKε, are activated through transautophosphorylation and subsequently phosphorylate the transcription factors, interferon regulatory factors 3 and 7 (10). Once phosphorylated, these interferon regulatory factors translocate to the nucleus to increase production of type I interferons, which will ultimately be exported out of the cell and bind to interferon receptors on neighboring cells through paracrine signaling (10, 11).

TBK1 and IKKε have been studied in several contexts outside of their innate immune regulatory roles. Mutations in TBK1 have been implicated in several central nervous system diseases, and TBK1 has been tied to autoimmune functions, where it regulates immune tolerance in dendritic cells by suppressing autoimmunity (12, 13). Likewise, IKKε has been shown to phosphorylate nuclear factor of activated T cells (NFATc1) as a mechanism hindering T-cell activation (14). Furthermore, TBK1 has been studied in the context of mitosis, with work associating it with the centrosome and identifying mitotic proteins as TBK1 substrates (15). In addition, both TBK1 and IKKε have been studied in the context of cancer (9, 16, 17, 18). IKKε expression has been shown to be elevated in pancreatic ductal adenocarcinoma (PDAC), as well as in gliomas (19, 20). The work of Rajurkar *et al.* (19) showed that IKKε promotes tumor growth using MIA PaCa-2 pancreatic cells in a xenograft model, linking IKKε activity with the promotion of nuclear levels of the transcription factor GLI1. Furthermore, IKKε expression in pancreatic cancer has been linked with reprogramming of glucose metabolism (21). Likewise, higher TBK1 protein expression has been linked with PDAC (22). It was reported that some KRAS mutant cancer cells displayed synthetic lethality with TBK1 knockdown (23). A separate report found that not all KRAS-dependent cells exhibit reliance on TBK1 (24). In cancer cells, TBK1 activity was shown to be regulated through the RalB–GTPase pathway, coupling innate immune signaling with cancer phenotypes (25). Consistent with this, TBK1 was shown to be activated in PDAC through an Axl–RalB pathway to promote epithelial plasticity (22). In addition, TBK1 was linked with immunosuppression in a KRAS-dependent lung tumor model (26). Despite their structural similarity, there has been limited research to explore whether TBK1 and IKKε function together to promote cancer-associated phenotypes.

To further explore oncogenic roles of KRAS and MAPK signaling, as well as investigate potential overlapping and distinct cancer-related roles of TBK1 and IKKε, we studied their expression in pancreatic cancer. We found that TBK1 and IKKε are more highly expressed in tumor tissues than in normal tissues, consistent with other groups’ findings. Our data indicate that RAS-induced signaling in pancreatic and other cancer cell lines controls IKKε levels, possibly through control of protein stability or translation and that this is mediated through ERK signaling. TBK1 levels are largely unaffected by ERK signaling, consistent with its control through the RalB pathway. Individual knockdown of either IKKε or TBK1 did not confer a significant growth defect in KRAS mutant MIA PaCa-2 pancreatic cancer cells, however, double knockdown reduced cell growth and induced cell death. Indications of the importance of TBK1 and IKKε downstream of ERK signaling are derived from RNA-Seq studies with evidence that TBK1–IKKε contribute to the regulation of a subset of genes controlled by ERK. Combining proteomic studies with RNA-seq data indicate that the expression of many proteins is regulated by ERK through stability and not RNA regulation, with many of these proteins associated with translation control. In addition, knockdown or inhibition of TBK1 and IKKε reduced tumor sphere growth, which is supported by loss of stemness signaling signatures with ERK inhibition and with TBK1–IKKε inhibition. Overall, the data suggest the importance of ERK regulation of IKKε, combined with roles for TBK1, in promoting oncogenic phenotypes associated with RAS-induced cancer cell signaling.

## Results

### TBK1 and IKKε protein levels are elevated in pancreatic tumors

**Figure 1:**
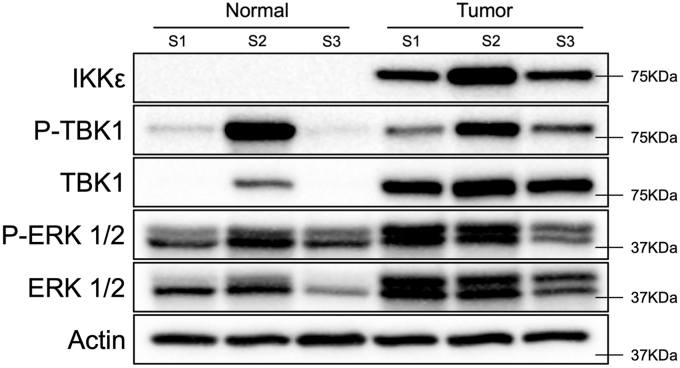
**TBK1 and IKKε exhibit elevated expression in pancreatic tumor tissues.** Immunoblotting for IKKε, TBK1, P-TBK1, P-ERK1/2, ERK1/2, and β-Actin in tissue obtained from adjacent pancreas (normal) and in pancreatic tumors maintained through PDX passage (tumor). A KRAS mutation status of G12V was confirmed by the donating laboratory for two of the three tumor samples: S1 and S3. See the Experimental procedures section for further description. ERK1/2, extracellular signal–regulated kinase 1/2; IKKε, inhibitor of nuclear factor kappa-B kinase epsilon; KRAS, Kirsten's rat sarcoma virus; TBK1, TANK-binding kinase 1.

Previous publications have implicated roles for TBK1 and IKKε in several cancer types, including work on pancreatic cell lines and tumor models (9). To explore this relationship further, we profiled expression of TBK1 and IKKε in pancreatic tumor–adjacent tissues relative to patient-derived pancreatic xenograft tumor samples. This analysis revealed an increase of IKKε and TBK1 protein levels in the tumor samples as compared with the normal tissues (Fig. 1). This increase of protein level correlated with ERK1/2 phosphorylation (P-ERK), a marker of KRAS and MAPK activity, which was expectedly elevated in the tumor samples. Interestingly, one of the tumor-adjacent samples exhibited elevated expression of TBK1 and phosphorylated TBK1 (P-TBK1), and this correlated with a modest elevation of P-ERK. These results suggest that TBK1 and IKKε protein expression levels are elevated in pancreatic cancer, potentially through active KRAS–MAPK signaling.

### KRAS, MEK, and ERK regulate IKKε and TBK1 protein levels in KRAS mutant cancer cells

**Figure 2:**
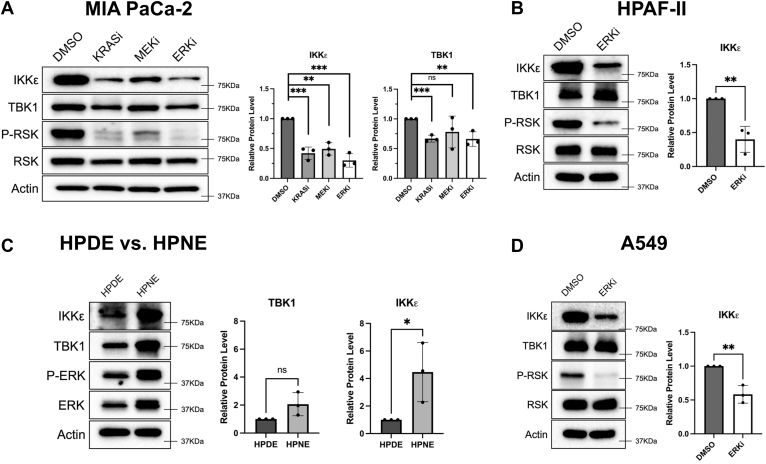
**Regulation of IKKε and TBK1 by KRAS and MAPK signaling in multiple cell lines.***A*, MIA PaCa-2 cells were treated with 10 μM ARS1620, 5 nM trametinib, or 1 μM SCH772984 for 24-h before harvest. Antibodies used were IKKε, TBK1, RSK, P-RSK, and β-Actin. Blots were quantified using Fiji (ImageJ) and normalized to β-Actin. An unpaired, parametric *t* test was used for each inhibitor to determine statistical significance. *IKKε*—(KRASi: ∗∗∗*p* = 0.0007, MEKi: ∗∗*p* = 0.0011, and ERKi: ∗∗∗*p* = 0.0004). *TBK1*—(KRASi: ∗∗∗*p* = 0.0005, ERKi: ∗∗*p* = 0.0087). *B*, HPAF-II cells were treated with 1 μM SCH772984 for 24-h before harvesting and immunoblotting as above. An unpaired, parametric *t* test was used to determine statistical significance. *IKKε*—(ERKi: ∗∗*p* = 0.0056). *C*, untreated HPDE and HPNE cells were collected and analyzed *via* immunoblotting with identical statistical analysis as described in *A* being performed on IKKε protein levels (∗*p* = 0.0496). *D*, A549 cells were treated with 1 μM SCH772984 for 24-h before harvesting and immunoblotting. An unpaired, parametric *t* test was used to determine statistical significance (ERKi: ∗∗*p* = 0.0052). All blots are representative of three biological replicates. All bar graphs represent mean values with standard deviation. HPDE, human pancreatic duct epithelial; HPNE, human pancreatic nestin-expressing; IKKε, inhibitor of nuclear factor kappa-B kinase epsilon; KRAS, Kirsten's rat sarcoma virus; MAPK, mitogen-activated protein kinase; TBK1, TANK-binding kinase 1.

Given the previously published connections of TBK1 and IKKε with cancer and the correlation with MAPK activity seen in Fig. 1, we explored TBK1 and IKKε regulation in the widely studied MIA PaCa-2 pancreatic cancer cell line, which expresses G12C mutant KRAS. To inhibit critical signaling proteins in pancreatic cancer, we used ARS1620 to target the G12C mutant KRAS, trametinib to target MEK, and SCH772984 for ERK1/2 inhibition, using phosphorylated RSK (P-RSK) as a marker of KRAS and MAPK activity. Individual 24-h treatment with these inhibitors led to a significant loss of IKKε; however, TBK1 protein levels displayed a less robust response (Fig. 2*A*). To determine if results would be similar in a different pancreatic cancer cell line, we repeated the 24-h SCH772984 treatment in HPAF-II pancreatic cancer cells, which express G12D mutant KRAS. As with the MIA PaCa-2 result, IKKε levels were reduced with ERK inhibition in HPAF-II cells; however, a loss of TBK1 was not observed (Fig. 2*B*). To explore this regulation outside the pancreatic cancer cell lines, human pancreatic cancer organoid cultures were treated with the same ERK inhibitor for 24-h, and a similar loss of IKKε expression was measured (Fig. S1). These results suggest that KRAS- and MAPK-regulated control of IKKε protein levels is common across pancreatic cancers, yet the regulation of TBK1 by this pathway is variable.

We then aimed to explore the possibility that the control of IKKε through ERK signaling would be found outside of oncogenic settings. To this end, we compared the expression of IKKε and TBK1 between human pancreatic duct epithelial (HPDE) and human pancreatic nestin-expressing (HPNE) immortalized pancreatic epithelial cells. Consistent with the hypothesis that ERK signaling increases IKKε and potentially TBK1 levels, higher ERK activity was observed in HPNE cells (represented by higher P-ERK levels), correlating with a significant increase in IKKε and an elevated, but not significant, increase in TBK1 protein levels (Fig. 2*C*). This suggests that ERK signaling, irrespective of oncogenic status, can promote IKKε levels. Finally, we aimed to determine if the effect on IKKε, and possibly TBK1, would be seen in KRAS-mutant, non-pancreatic cancer cells. We therefore performed 24-h SCH772984 treatment of A549 KRAS G12S mutant lung cancer cells, where we observed a loss of IKKε, but not TBK1 protein levels (Fig. 2*D*). Taken together, these results suggest that the MAPK-regulated control of IKKε occurs across immortalized pancreatic epithelial cells, different cancer cell lines, and human pancreatic cancer (KRAS mutant) organoids.

Given that our data indicated that ERK controls IKKε protein levels, we aimed to explore whether IKKε or TBK1 affect ERK1/2 expression and/or activity. We performed knockdown of TBK1 and/or IKKε in MIA PaCa-2 cells and analyzed ERK activity through its level of phosphorylation as measured by immunoblotting. Knockdown of IKKε (individually as well as in the dual knockdown), but not knockdown of TBK1, enhanced ERK phosphorylation (Fig. S2). These results suggest that ERK activation of IKKε functions to limit ERK activity in KRAS-driven MIA PaCa-2 cells.

### MAPK-controlled regulation of IKKε is post-transcriptional

**Figure 3:**
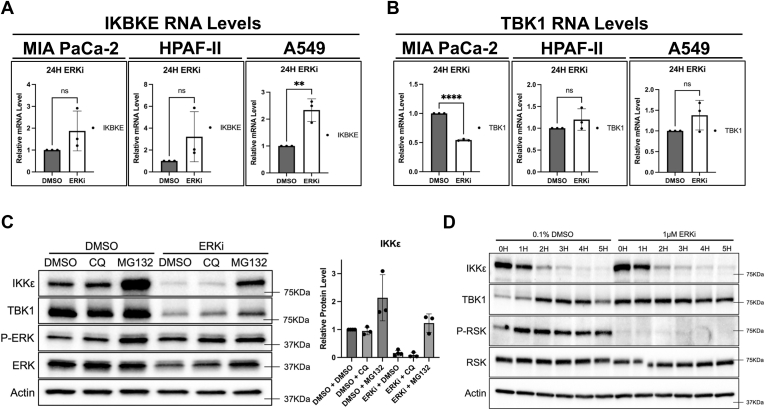
**MAPK regulation of IKKε occurs at the protein level.***A* and *B*, MIA PaCa-2, HPAF-II, or A549 cells were treated with 1 μM SCH772984 for 24-h before RNA purification and qPCR analysis for IKBKE (*A*) or TBK1 (*B*). Results were normalized to GUSB, and relative quantities were determined using ΔCT values. Unpaired, parametric *t* tests were used to determine statistical significance (A549 IKKε: ∗∗*p* = 0.0051), (MIA PaCa-2 TBK1: ∗∗∗∗*p* < 0.0001). *C*, MIA PaCa-2 cells were treated for 24-h with 1 μM SCH772984, with the culture medium being supplemented with 0.1% dimethyl sulfoxide, 10μM chloroquine, or 1μM MG132 at 12-h. Cells were harvested and analyzed *via* immunoblotting. Western blot band intensity was determined using Fiji (ImageJ) and graphed using Prism. All bar graphs represent mean values with standard deviation. *D*, MIA PaCa-2 cells were treated for 4-h with 1μM SCH772984, at which point the medium was supplemented with 100 μg/ml cycloheximide. Samples were harvested at 1-h intervals. All blots are representative of three biological replicates. IKKε, inhibitor of nuclear factor kappa-B kinase epsilon; MAPK, mitogen-activated protein kinase; qPCR, quantitative PCR; TBK1, TANK-binding kinase 1.

Having determined that IKKε and, inconsistently, TBK1 protein levels are reduced under KRAS or MAPK inhibition, we then probed the level of this regulation. Using quantitative PCR, we quantified IKKε (IKBKE) and TBK1 mRNA *via* normalization to a GUSB internal control in different KRAS mutant cancer cells. MIA PaCa-2, HPAF-II, and A549 cells each exhibited an increase in IKBKE mRNA levels following ERK inhibition, opposite to that of the effect of the inhibitor on protein levels (Fig. 3*A*). The increase was significant in A549 cells, but not in MIA PaCa-2 or HPAF-II cells, suggesting an ERK-dependent regulatory mechanism on IKKε expression independent of mRNA levels. MIA PaCa-2 cells displayed a significant loss of TBK1 mRNA (Fig. 3*B*) paralleling the loss of protein with ERK inhibition (Fig. 2*A*). However, we noted no significant increase or decrease of TBK1 mRNA levels in HPAF-II or A549 cells, overall suggesting that the regulation of TBK1 is distinct from that of IKKε.

We then explored the regulation of IKKε at the protein level. To this end, we treated MIA PaCa-2 cells with SCH772984 for 12-h before supplementing the media with chloroquine or MG132 to block potential autophagy- or proteasome-mediated effects, respectively. We found that MG132 was able to partially block the loss of IKKε induced by MAPK inhibition, whereas chloroquine was unable to do so (Fig. 3*C*). Interestingly, MG132 treatment increased IKKε levels independent of SCH772984 treatment. This result suggests that IKKε turnover is robust and directed through the proteasome rather than the autophagosome. To explore protein turnover of IKKε and to determine if ERK inhibition decreases IKKε half-life, MIA PaCa-2 cells were treated for 4-h with SCH772984 before adding 100 μg/ml cycloheximide to block translation. Cells were harvested at 1-h intervals to generate extracts to be used in immunoblotting. Results from this experiment show that the half-life of IKKε in MIA PaCa-2 cells is approximately 1-h and, in contrast, TBK1 is highly stable (Fig. 3*D*). ERK inhibition did not further reduce IKKε half-life within the time frame of the study (Fig. 3*D*).

### Analysis of IKKε RNA levels relative to protein levels from the Clinical Proteomic Tumor Analysis Consortium database

To determine if IKKε mRNA levels correlate with pancreatic cancer patient survival, we generated a Kaplan–Meier plot from The Cancer Genome Atlas pancreatic cancer database. This analysis demonstrates that elevated IKKε mRNA levels are associated with poorer survival in pancreatic cancers (Fig. S3*A*). Given the lack of direct correlation between mRNA levels and protein loss seen across several cancer cell lines with ERK inhibition (Figs. 2 and 3), we used Clinical Proteomic Tumor Analysis Consortium data to compare the RNA and protein expression of IKKε in pancreatic cancer patients. This analysis revealed a moderate correlation between IKKε RNA and protein levels in some tumors, whereas others lacked any distinct correlation between protein and RNA (Fig. S3*B*). These data underscore the concept that survival data generated from mRNA levels may be incomplete and might not take into account protein levels and/or protein activity. The results also suggest that, in some tumors, additional mechanisms are involved in the regulation of IKKε protein levels distinct from being directly promoted by mRNA levels.

### Transcriptomic and proteomic profiling with ERK and TBK1–IKKε inhibition

**Figure 4:**
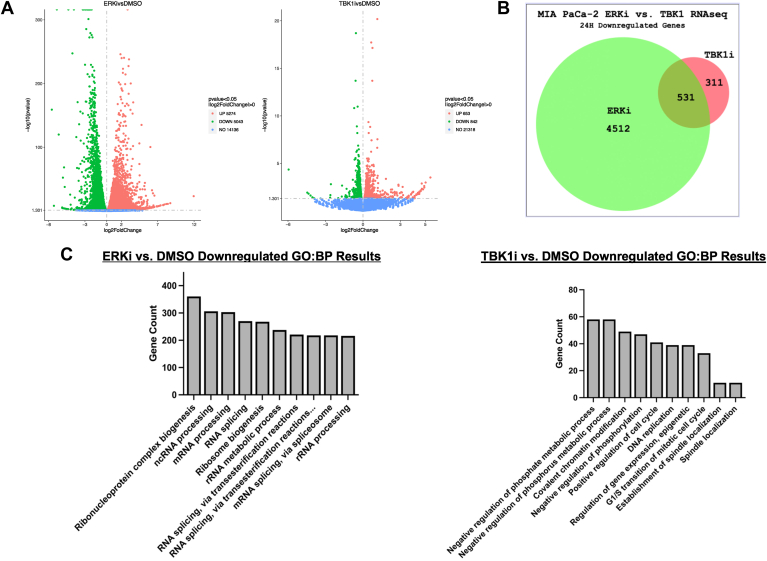
**Transcriptomic profiling reveals overlapping regulation of genes by TBK1–IKKε and ERK.***A*, MIA PaCa-2 cells were treated for 24-h with 1μM SCH772984 or 1μM compound 1 before RNA purification and quantification. RNA-Seq was performed by Novogene. Volcano plots were generated by Novogene using significant (*p* < 0.05) differentially expressed genes. *B*, a Venn diagram was generated using BioVenn presenting differentially expressed genes that were downregulated with ERK inhibition or TBK1 (*p* < 0.05). *C*, Gene Ontology analysis was performed by Novogene displaying biological processes (GO:BP) associated with the differentially expressed downregulated gene sets in *A*. ERK, extracellular signal–regulated kinase; IKKε, inhibitor of nuclear factor kappa-B kinase epsilon; TBK1, TANK-binding kinase 1.

To explore potential overlapping regulation of genes and oncogenic signaling pathways through ERK and TBK1–IKKε in pancreatic cancer, we treated MIA PaCa-2 cells for 24-h with the ERK inhibitor SCH772984 or the TBK1–IKKε inhibitor compound 1 (27) before RNA purification and RNA-Seq analysis. This RNA-Seq analysis identified a large number of genes (10,317 genes) significantly differentially expressed with ERK inhibition (5274 upregulated and 5043 downregulated) (Fig. 4*C* shows downregulated analysis), a finding consistent with previous publications implicating ERK as a major regulator in pancreatic cancer signaling (6, 7). Using the TBK1–IKKε inhibitor, 842 genes were significantly downregulated in MIA PaCa-2 cells with an overlap of 531 genes with the ERK-inhibited gene set (Fig. 4*B*). Analysis of the downregulated genes in cells treated with SCH772984 revealed processes associated with ribosomes and translation, cell division, and RNA mechanisms. With compound 1 treatment, downregulated genes were associated with DNA replication, cell division, and adhesion (Fig. 4*C*). A Venn diagram depicts genes downregulated with ERK inhibition, TBK1–IKKε inhibition, and the overlap of genes downregulated by both inhibitors (Fig. 4*B*).

We explored global proteomics associated with ERK inhibition in MIA PaCa-2 cells, which led to downregulation/upregulation of a number of proteins (Fig. S4*A*). IKKε was not detected basally or differentially regulated in this LC–MS analysis. This could be due to low abundance of IKKε or higher levels of other proteins keeping IKKε below the detection limit of the instrument. For proteins downregulated by ERK inhibition, a large portion did not correlate with downregulation of RNA as related to the aforementioned RNA-Seq analysis (Fig. S4*B*). This result suggests that ERK controls the stability of a large number of proteins in MIA PaCa-2 cells, either translationally or post-translationally. Biological processes of proteins lost with ERK inhibition and not affected at the RNA level are associated with translation and translation-associated mechanisms (Fig. S4*C*).

### Loss of both TBK1 and IKKε confers a growth defect in MIA PaCa-2 cells

**Figure 5:**
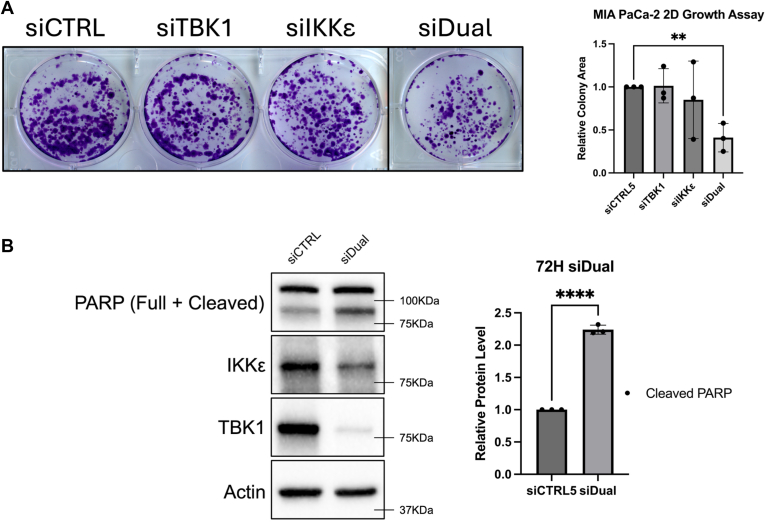
**Knockdown of TBK1 and IKKε confers a growth defect in MIA PaCa-2 cells.***A*, MIA PaCa-2 cells were plated at a density of 5000 cells/well on a 6-well plate before treatment with siRNA targeting TBK1 and/or IKKε. Nontargeting siRNA was used as a control and was added to the individual knockdowns to ensure the total RNA concentration was equivalent across conditions. Plates were grown for 1 to 2 weeks before staining with crystal violet. A single image was spliced as indicated by the *black* border around well number 4 to keep all treated wells in line. Images were quantified using the colony area and colony measure features in Fiji (ImageJ). Area values were compared using an unpaired *t* test for each siRNA (∗∗*p* = 0.0035). *B*, MIA PaCa-2 cells were treated for 72-h with siRNA targeting both TBK1 and IKKε (siDual). Band intensity of cleaved PARP was determined using Fiji (ImageJ) and normalized to β-Actin. Statistical analysis was performed using an unpaired, parametric *t* test in GraphPad Prism (*∗∗∗∗p* < 0.0001). All blots and images are representative of three biological replicates. IKKε, inhibitor of nuclear factor kappa-B kinase epsilon; PARP, poly (ADP-ribose) polymerase; TBK1, TANK-binding kinase 1.

To study the effects of TBK1 and IKKε on pancreatic cancer cell growth, we plated MIA PaCa-2 cells at a low seeding density on 6-well cell culture plates before treatment with siRNA targeting TBK1, IKKε, or both kinases (siDual). The cells were allowed to grow for ∼2 weeks, with siRNA-free growth medium being refreshed every 2-3 days. Cells were stained with crystal violet before quantification. Knockdown of TBK1 or IKKε alone did not lead to a growth defect of MIA PaCa-2 cells, whereas knockdown of both kinases imparted an approximate 60% decrease in cell growth using this assay (Fig. 5*A*). We then aimed to determine if cell death occurred because of IKKε and TBK1 loss. MIA PaCa-2 cells were treated with siRNA targeting both kinases for 72-h. This triggered a significant increase in cleaved PARP (poly (ADP-ribose) polymerase), a marker of apoptosis (Fig. 5*B*). These results suggest that together, but not separately, IKKε and TBK1 control growth and survival of MIA PaCa-2 pancreatic cancer cells.

### TBK1 and IKKε promote cancer cell stemness in pancreatic cancer cells

**Figure 6:**
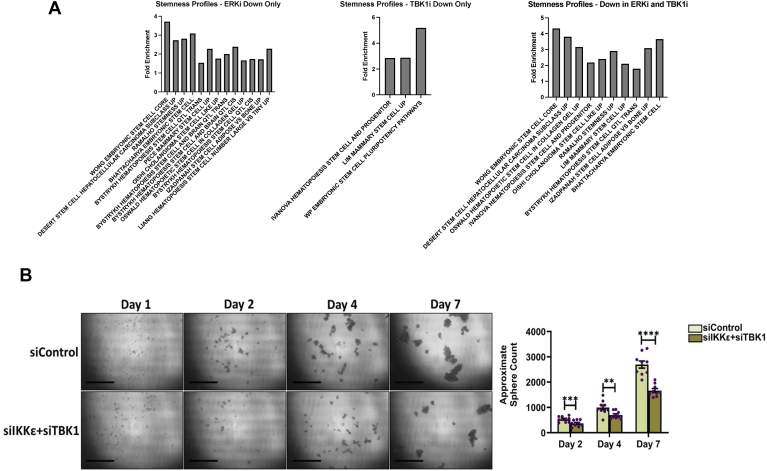
**Dualknockdown of TBK1 and IKKε decreases MIA PaCa-2 tumor sphere formation.***A*, differentially expressed genes that were downregulated with TBK1–IKKε inhibition and/or ERK inhibition in MIA PaCa-2 cells were used to perform enrichment signature analysis using MSigDB-curated gene sets related with cancer stem cells (false discovery rate <0.05). *B*, siControl and siTBK1/siIKKε-treated MIA PaCa-2 cells were seeded for tumor sphere assays and images were taken on days 1, 2, 4, and 7. Quantification of tumor sphere formation was performed on days 2, 4, and 7 (∗∗*p* = 0.011370, ∗∗∗*p* = 0.009744, and ∗∗∗∗*p* < 0.0001) (n = 3, and each replicate was seeded in three wells). Scale bar represents 100 μm. Data were analyzed using unpaired multiple *t* test. ERK, extracellular signal–regulated kinase; IKKε, inhibitor of nuclear factor kappa-B kinase epsilon; TBK1, TANK-binding kinase 1.

Additional analysis of the RNA-Seq data described previously (Fig. 4) demonstrated that ERK inhibition of MIA PaCa-2 cells led to a reduction in pathways associated with stemness and cancer stem cells. Treatment with compound 1 (IKKε and TBK1 inhibitor) also reduced stemness-related pathways (Fig. 6*A*). Genes downregulated with both ERK and TBK1–IKKε inhibitors related to stem cells include CDC25A, TRIM28, HNRNPU, and MTOR (28, 29). To determine if TBK1 and IKKε contribute to a cancer stemness phenotype, we performed tumor sphere assays using MIA PaCa-2 cells. MIA PaCa-2 cells were transfected with siRNA targeting both TBK1 and IKKε before plating on low-adhesion cell culture plates following our previous protocol (30). Cells were imaged at the 24, 48, 96, and 168-h marks with tumor spheres larger than 60 μm being counted. Results demonstrate that knockdown of TBK1 and IKKε caused a significant loss of sphere formation (Fig. 6*B*). We also noted a similar loss of sphere formation in MIA PaCa-2 cells when treated with the TBK1–IKKε inhibitor, compound 1 (Fig. S5). These findings are consistent with a reduction of cancer cell stemness pathways and related genes found in the RNA-Seq studies. This suggests that IKKε and TBK1, together, promote an environment for cancer stem cell formation and maintenance in pancreatic cancer cells, thereby contributing to aggressiveness.

## Discussion

In pancreatic cancer cells, as well as other cancer cells, RAS signaling is often a critical driver of oncogenic phenotypes (2, 3). RAS activation, *via* mutation or through growth factor receptor stimulation, leads to activation of downstream effector signaling pathways, including the activation of ERK1/2 driven by upstream RAF and MEK activation (3). The importance of ERK signaling in cancer is underscored by the utilization of ERK inhibitors in the clinic, which have shown promise (5). Specific mechanisms and targets downstream of ERK that contribute to cancer phenotypes are limited (see *Introduction* section) but can include phosphorylation of certain transcription factors as well as the mitochondrial protein Drp1 (31). Here, we present evidence that ERK signaling regulates the protein levels of the IKK-family kinase IKKε. We find that this regulation occurs in different cancer cells as well as organoid models, with limited effects on TBK1 (Figs. 2 and S1). However, both TBK1 and IKKε contribute to growth and survival of RAS-mutant MIA PaCa-2 cells (Figure 5, Figure 6 and S5). This is consistent with evidence that TBK1 activity is regulated by the RAL pathway downstream of RAS (25), thus ERK signaling to promote IKKε levels and RAL signaling through TBK1 may underlie differential regulation of both TBK1 and IKKε to contribute to the oncogenic effects of mutant RAS.

To address mechanisms related to the effects of ERK signaling on IKKε protein levels (Fig. 2), we found that IKKε RNA levels were not reduced in response to ERK inhibition across several cell lines (Fig. 3). Further analysis showed that the half-life of IKKε in MIA PaCa-2 cells is approximately 1-h, whereas TBK1 is highly stable, indicating distinct modes of regulation of these related kinases. Furthermore, ERK inhibition did not reduce the basal half-life of IKKε indicating the downregulation promoted by ERK inhibition is a slower mechanism (Fig. 3). Proteasome inhibition stabilizes both basal and ERK-induced levels of IKKε, although the mechanism underlying this effect is not understood. These findings strongly suggest that the regulation of IKKε by KRAS–MAPK signaling is occurring through translational control. This hypothesis is supported by the proteomic profiling indicating a unique difference between the effects of ERK inhibition on the proteasome and the transcriptome (Fig. S4). Future studies would address whether translational control or potential degron-mediated mechanisms promote loss of IKKε following ERK inhibition. These mechanisms have been linked with ERK signaling in earlier studies (32, 33, 34). Interestingly, while ERK inhibition reduces IKKε expression, knockdown of IKKε led to an increase in ERK activity (Fig. S2). Thus, a function for IKKε downstream of RAS and ERK may be to limit ERK activity, which has the potential to lead to cell death when chronically activated (35).

To gain insight into potential roles for IKKε and TBK1 downstream of ERK signaling, we performed RNA-Seq of MIA PaCa-2 cells with ERK inhibition and with inhibition of TBK1 and IKKε. ERK inhibition led to large changes in gene expression in MIA PaCa-2 cells, with many genes downregulated or upregulated. Compound 1, an established TBK1 and IKKε inhibitor, less robustly altered gene expression, though overlap with the ERK inhibitor–treated samples was clearly noted. ERK inhibition reduced expression of genes associated with ribosome biogenesis and protein translation, whereas TBK1–IKKε inhibition triggered effects on cell cycle and mitosis. ERK is known to positively regulate several transcription factors, and TBK1 and IKKε are also implicated in controlling transcription factors such as NF-κB (9). These results (Fig. 4) suggest that IKKε and/or TBK1 mediate effects on gene expression that are initiated by upstream ERK signaling.

Both TBK1 and IKKε have been linked with pancreatic cancer cell phenotypes. In our analysis of MIA PaCa-2 cells, individual knockdown of TBK1 or IKKε did not affect cell growth/survival; however, dual knockdown significantly suppressed cell growth, which is linked with induction of cell death (Fig. 5). These results suggest that TBK1 and IKKε control separate mechanisms that, together, drive this oncogenic phenotype. Long-term studies are needed to identify potential substrates for TBK1 and/or IKKε that contribute to their phenotypic effects in pancreatic cancer cells. Given their related sequences, there may be common, but also likely unique, targets leading to their combined effects on cell survival. The potential for kinase-independent effects of TBK1 or IKKε also remain.

Further analysis of the RNA-Seq pathway data revealed that pathways involved in cancer stem cell mechanisms were lost with both ERK inhibition and with the TBK1–IKKε inhibitor. We found that either knockdown or inhibition of TBK1 and IKKε reduced tumor sphere number and size in MIA PaCa-2 cells (Figs. 6 and S5), potentially overlapping with effects on cell survival. Specific genes commonly affected by ERK inhibition and TBK1–IKKε inhibition such as TRIM28 have been linked with stem cells and other cancer-associated mechanisms (36). These data further support the hypothesis that TBK1 and IKKε function together in the RAS signaling pathway to promote oncogenic phenotypes, for which the regulation of IKKε by ERK is a critical component.

## Experimental procedures

### Cell culture

A549 and HPAF-II cell lines were obtained from the UNC Tissue Culture Facility, MIA PaCa-2 cells were obtained from the American Type Culture Collection, and HPDE and HPNE cells were a generous gift from Kirsten Bryant, PhD in the Lineberger Comprehensive Cancer Center at UNC Chapel Hill. HPDE and HPNE cells were cultured at 5% CO_2_ and 37 °C in Dulbecco’s modified Eagle's medium (DMEM; Gibco; catalog no.: 11995-065) supplemented with 10% fetal bovine serum (FBS) and 1× penicillin–streptomycin (Corning; catalog no.: 30-002-CI). A549 cells were maintained in F-12 medium (Gibco; catalog no.: 11765-054) with a supplementation of 10% FBS and 1× penicillin–streptomycin. HPAF-II cells were maintained in Eagle’s minimum essential medium (Corning; catalog no.: 10-010-CV) supplemented with 1× Nonessential Amino Acid Cell Culture Supplement (Gibco; catalog no.: 11140-050), 10% FBS, and 1× penicillin–streptomycin. MIA PaCa-2 Cells were cultured in DMEM (Gibco; catalog no.: 11995-065) with 10% FBS, 2.5% horse serum (Gibco; catalog no.: 16050-122), and 1× penicillin–streptomycin. Cells were passaged less than 15 times from thawing. MIA PaCa-2 cells were validated with short tandem repeat analysis and tested negative for mycoplasma contamination. Organoids were generated and maintained as previously described (37).

### Patient-derived xenograft samples:

Primary tumor samples were generous gifts from the Yeh Lab at UNC Chapel Hill and were obtained as described previously (38). Briefly, primary tumor samples were obtained from the UNC Tissue Procurement Core Facility adhering to all necessary Institutional Review Board regulations and guidelines. The “normal” samples were collected directly from patients in regions of the pancreas adjacent to the tumor. Patient tumor samples were subcutaneously implanted into immunocompromised mice before harvest and lysis as described later. Samples are not matched.

### Antibodies and reagents

Antibodies were diluted in 5% bovine serum albumin (BSA)/1× Tris-buffered saline with 0.1% Tween-20 (TBST). Antibodies targeting IKKε (CST; catalog no.: 3416), TBK1 (CST; catalog no.: 38066), P-TBK1 S172 (CST; catalog no.: 5483), P-ERK1/2 T302/Y204 (CST; catalog no.: 4370), ERK1/2 (CST; catalog no.: 4696), p-RSK S363/T359 (CST; catalog no.: 9344), RSK1/2/3 (CST; catalog no.: 9355), PARP (CST; catalog no.: 9542), and β-Actin (CST; catalog no.: 3700) were purchased from Cell Signaling Technologies. IKKε and TBK1 antibody specificity was demonstrated using siRNA (Supporting Information). Pathway manipulation with small-molecule inhibitors demonstrated that P-RSK antibodies were specific to phosphorylated targets. Small-molecule inhibitors were purchased from SelleckChem (SCH772984: S7101, trametinib: S2673, and ARS1620: S8707). The TBK1–IKKε inhibitor, compound 1 (27) was a generous gift from the Qing Zhang, PhD lab at UT Southwestern. Cycloheximide was purchased from Sigma (C-7698), and MG132 was purchased from EMD Millipore (catalog no.: 474790-5MG).

### RNA interference

Dharmacon SMARTPool siRNA was purchased from Horizon Discovery targeting IKKε (catalog no.: M-003723-02-0005), TBK1 (catalog no.: M-003788-02-0005), or a nontargeting control (catalog no.: D-001210-05-05). siRNA was resuspended in 5× siRNA Buffer (Horizon; catalog no.: B-002000-UB-100) diluted to 1× with water. siRNA was transfected into cells using DharmaFECT 1 (Horizon; catalog no.: T-2001-01) and Opti-Mem I (Gibco; catalog no.: 31985070).

### Immunoblotting

Cells were washed three times on ice in their respective culture plate with cold 1× phosphate-buffered saline (Corning; catalog no.: 21-031-CV) before scraping into microcentrifuge tubes and brief centrifugation to pellet whole cells. Cell pellet was resuspended in radioimmunoprecipitation assay lysis buffer (Boston BioProducts; catalog no.: BP-115) supplemented with 1× HALT, 1× EDTA (Thermo; catalog no.: 78438), and 2× Phosphatase Inhibitor Cocktail 3 (Sigma; catalog no.: P0044). Samples were incubated on ice for 5 min before centrifugation at 16,100 rcf for 10 min at 4 °C.

Tumor-adjacent and PDX tumor samples were added to tubes containing ceramic beads and 500 μl of radioimmunoprecipitation assay lysis buffer supplemented with 10× HALT, 10× EDTA, and 10× phosphatase inhibitor. Samples were lysed on ice for 10 min before agitation for 5 min at 4 °C. Samples were sonicated using a probe sonicator (3 × 10-s at 30% amplitude) with time to cool between sonication runs. Samples were transferred to a new tube and clarified using a centrifuge at 16,100 rcf for 10 min at 4 °C.

The supernatant was used to quantify protein amounts using the Bradford Protein Determination Assay (Bio-Rad; catalog no.: 5000006). Samples were then diluted to their working concentrations with 4× Laemmli buffer (Bio-Rad; catalog no.: 161-0747). Samples were boiled at 98 °C for 5 min and loaded onto gels (Bio-Rad; catalog nos.: 4568084, 4561085, 4568086, 5671084, or 5671085). Gels were transferred to 0.2 micron nitrocellulose membranes using the Bio-Rad Turbo Transfer system before transfer efficiency was verified using Ponceau stain (Sigma–Aldrich; catalog no.: P7170-1L). Membranes were blocked at room temperature for 1-h in 5% BSA (GoldBio; catalog no.: A-420-500) suspended in TBST. Membranes were incubated overnight at 4 °C in primary antibodies diluted in 5% BSA–TBST. Membranes were washed with TBST before incubation for 1-h in 5% milk/TBST with either rabbit or mouse secondary antibodies (Promega; catalog nos.: W4011 or W4021). Membranes were washed in TBST before imaging using the ChemiDoc/ImageLab imaging system and ECL reagent (Bio-Rad; catalog no.: 1705061). Band intensity was calculated using Fiji (ImageJ) and normalized to the respective control. Values were plotted, and statistics were run using GraphPad Prism (version 10.4.1; GraphPad Software, Inc).

### Quantitative PCR

RNA was isolated from cells using the Zymogen Quick-RNA MiniPrep Kit (Zymogen; catalog no.: 11-328) following the manufacturer’s suggested protocol. RNA quantification was performed using a NanoDrop. Complementary DNA (cDNA) was prepared using the Bio-Rad iScript cDNA synthesis kit (Bio-Rad; catalog no.: 1708891) and suggested thermocycler protocol. cDNA was analyzed using TaqMan probes (TBK1: Hs00179410_m1; IKKε/IKBKE: Hs01063858_m1; or GUSB: Hs99999908_m1) on QuantStudio6 or QuantStudio7 instruments. Analysis of cycle threshold values was performed using Microsoft Excel and plotted using GraphPad Prism before statistical analysis. Statistical analysis was performed using unpaired parametric *t* tests. *p* Values <0.05 were considered significant.

### Tumor sphere assay

Tumor sphere assay was performed as previously described (30). Briefly, MIA PaCa-2 cells were plated at a density of 3000 cells per well on 6-well ultralow adherent plates, using serum-free DMEM:F12 medium supplemented with 10 ng/ml basic fibroblast growth factor (Gibco; catalog no.: 100-18B-50UG), 20 ng/ml epidermal growth factor (Gibco; catalog no.: AF-100-15-100ug), and B-27 supplement (Life Technologies; catalog no.:17504044). Cells were incubated at 37 °C with 5% CO_2_ for 7 days, with images being taken at the noted days. Tumor spheres larger than 60 μm were counted on days 2, 4, and 7 for siRNA experiments and on days 2 and 4 for compound 1-treated cells.

### Global proteomics

#### Sample preparation

Samples were washed four times with cold PBS on their respective growth plates before scraping into microcentrifuge tube and brief centrifugation to pellet cells. PBS supernatant was aspirated, and cells were lysed in 8 M urea, 50 mM Tris–HCl (pH 7.8), 1× HALT, 1× EDTA, 1× Phosphatase Inhibitor Cocktail 3. Samples were lysed for 10 min on ice before probe sonication (two times for 5-s at 40% amplitude) on ice. Lysates were spun down at 16,100 rcf for 15 min at 4 °C before the supernatant was saved and quantified using the Bradford Protein Determination Assay (Bio-Rad). Quantified samples were stored at −80 °C until submission to the UNC MAP Core. Protein lysates were precipitated with acetone before resuspension in 1 M urea and 50 mM ammonium bicarbonate. Resuspended samples were reduced with 5 mM DTT and alkylated with 15 mM iodoacetamide for 45 min at room temperature. Samples were subjected to digestion with LysC (Wako) at 37 °C for 2-h and trypsin (Promega) overnight at 37 °C at a 1:50 enzyme:protein ratio. The resulting peptides were acidified with 0.5% trifluoroacetic acid and desalted using Thermo desalting spin columns. Eluates were dried *via* vacuum centrifugation, and peptide concentration was determined *via* Pierce Quantitative Fluorometric Assay. All samples were normalized to 0.1 μg/μl. A pooled sample was created by combining an aliquot of each sample.

#### LC–MS/MS analysis

All samples were spiked with iRT standard peptides (Biognosys) prior to LC–MS/MS analysis. Samples were then analyzed *via* LC–MS/MS using an Easy nLC 1200 coupled to a Fusion Lumos mass spectrometer (Thermo). The pooled sample was injected at the beginning and end of the sequence to assess technical reproducibility. Samples were injected onto an Easy Spray C18 column (75 μm id × 25 cm, 2 μm particle size; Thermo Scientific) and separated over a 122 min method. The gradient consisted of 5 to 45% mobile phase B at a 250 nl/min flow rate, where phase A was 0.1% formic acid in water and mobile phase B was 0.1% formic acid in 80% acetonitrile. The Lumos was operated in data-independent acquisition mode. A full MS scan (350-1200 *m/z*) was collected; resolution set to 120,000 with a maximum injection time of 45 ms and automatic gain control target of 250%. Following full MS scan, product ion scan was collected at a resolution of 30,000, with higher collision dissociation set to 30, automatic gain control target set to 2000%, maximum injection time to set to 54 ms, and 31 *m/z* precursor isolation windows.

#### Data analysis

Raw data files were processed using Spectronaut (v17; Biognosys) and searched against the UniProt reviewed human database (UP000005640, containing 20,404 entries, downloaded January 2023) and the MaxQuant common contaminants database (246 entries). The following settings were used: enzyme specificity set to trypsin, up to two missed cleavages allowed, cysteine carbamidomethylation set as a fixed modification, and methionine oxidation and N-terminal acetylation set as variable modifications. Precision iRT calibration was enabled. A false discovery rate of 1% was used to filter all data. Imputation was disabled, and single hit proteins were excluded. Unpaired Student’s *t* tests were conducted and *p* values, false discovery rate–corrected *p* values (*q* values), along with log2 fold change ratios were calculated in Spectronaut. Proteins with an absolute log2 fold change ≥0.6 and a *q* value (corrected *p* value) <0.05 were considered significant.

### RNA sequencing

MIA PaCa-2 cells were treated with 1 μM compound 1, 1 μM SCH772984, or dimethyl sulfoxide for 24-h before RNA extraction and quantification using the Zymogen Quick RNA Prep Kit. Samples were provided to Novogene for sequencing and analyzed using their previously described strategy (https://www.novogene.com/us-en/services/research-services/transcriptome-sequencing/mrna-sequencing/). Differentially expressed genes were compared using BioVenn (39).

### Survival and pathway analysis

Survival curve was generated using the Kaplan–Meier Plotter website (https://kmplot.com/analysis/). Gene sets from MSigDB and relevant publications were screened for keywords of interest pertaining to pancreatic cancer, including stemness. A list of gene sets of interest was then used for enrichment analysis performed using ShinyGO, version 0.77 (40).

### Gene and protein expression analysis

Clinical Proteomic Tumor Analysis Consortium PDAC data (2021) were retrieved from cBioPortal (41). RNA expression and protein abundance ratio corresponding to IKKε expression used in this study were median-centered *Z* scores as provided for all tumors. Tumors whose RNA and/or protein expressions were missing were excluded. GraphPad Prism (version 10.1.0) was used to plot the protein and RNA expression as a double gradient heat map.

## Data availability

Data referenced are available from the corresponding author upon request or are available at the locations listed below.

RNA-Seq fastq files and gene counts were uploaded to Gene Expression Omnibus: GSE289759.

The mass spectrometry proteomics data have been deposited to the ProteomeXchange Consortium *via* the PRIDE partner repository (42) with the dataset identifier: PXD060883.

## Supporting information

This article contains supporting information.

## Conflict of interest

The authors declare that they have no conflicts of interests with the contents of this article.
