# Supplementary material for: ERK signaling promotes IKKε expression and oncogenic functions in pancreatic cancer cells in association with TBK1
Source: J Biol Chem. 2025 Jul 28;301(9):110535. doi: 10.1016/j.jbc.2025.110535 (PMC12406268; doi:10.1016/j.jbc.2025.110535)
Supplement: Supporting Figures [file mmc1.pdf]

ERK signaling promotes IKKε expression and oncogenic functions in pancreatic cancer cells in association with TBK1

Adam Graves[1], Angana Mukherjee[2], Runying Yang[2], Angie Mordant[3], Thomas Webb[3], Kirsten Bryant[1,2], Laura Herring[3], Albert Baldwin[2]

From the [1]Department of Pharmacology, University of North Carolina at Chapel Hill, [2]Lineberger Comprehensive Cancer Center, and the [3]UNC Michael Hooker Metabolomics and Proteomics Core, University of North Carolina at Chapel Hill.

Material Included: Supporting figures 1 - 5

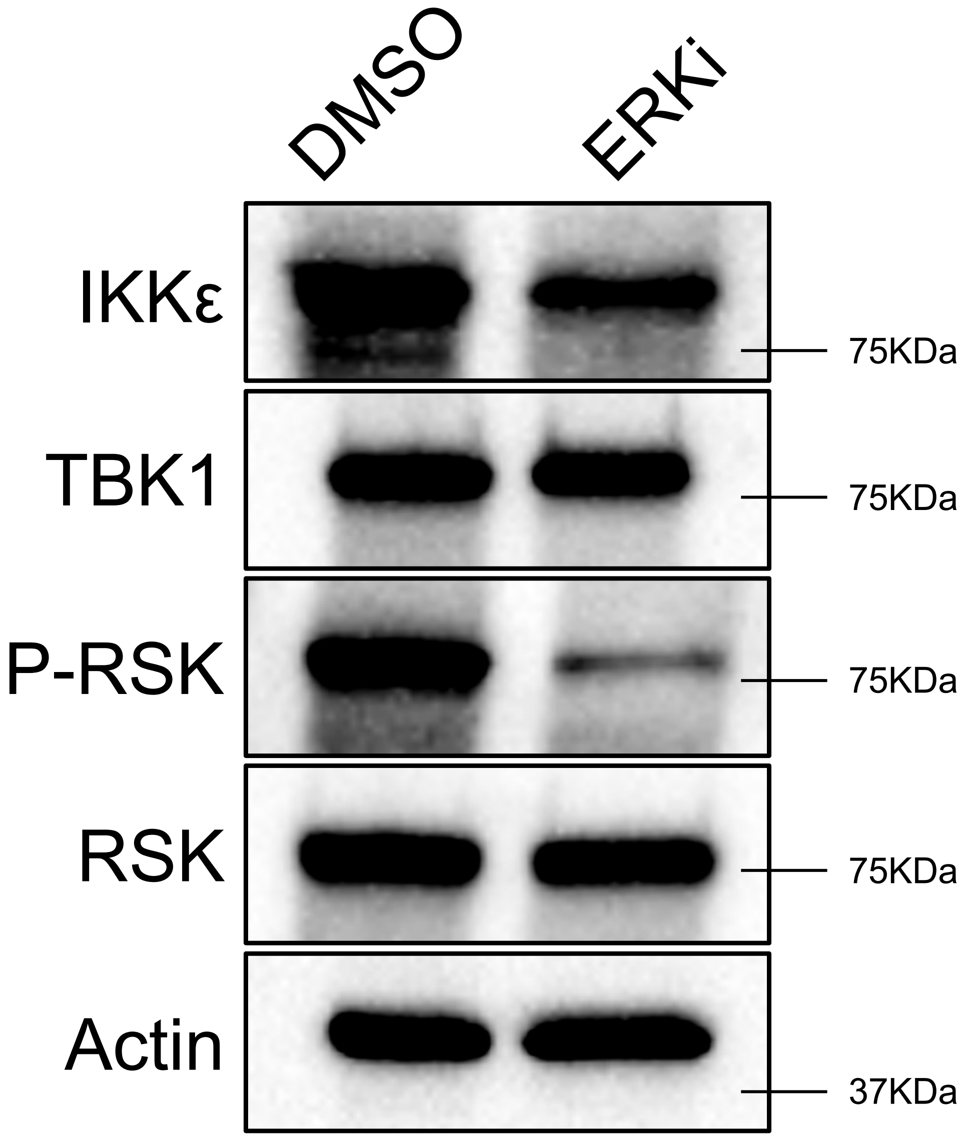

Supporting Information Figure 1 - ERKi-Induced Loss of IKKε is Present in Organoid Models

Organoid models were treated with SCH772984 for 24 hours before harvest. Blots are representative of three replicates of organoids containing either KRAS G12V or G12D mutations.

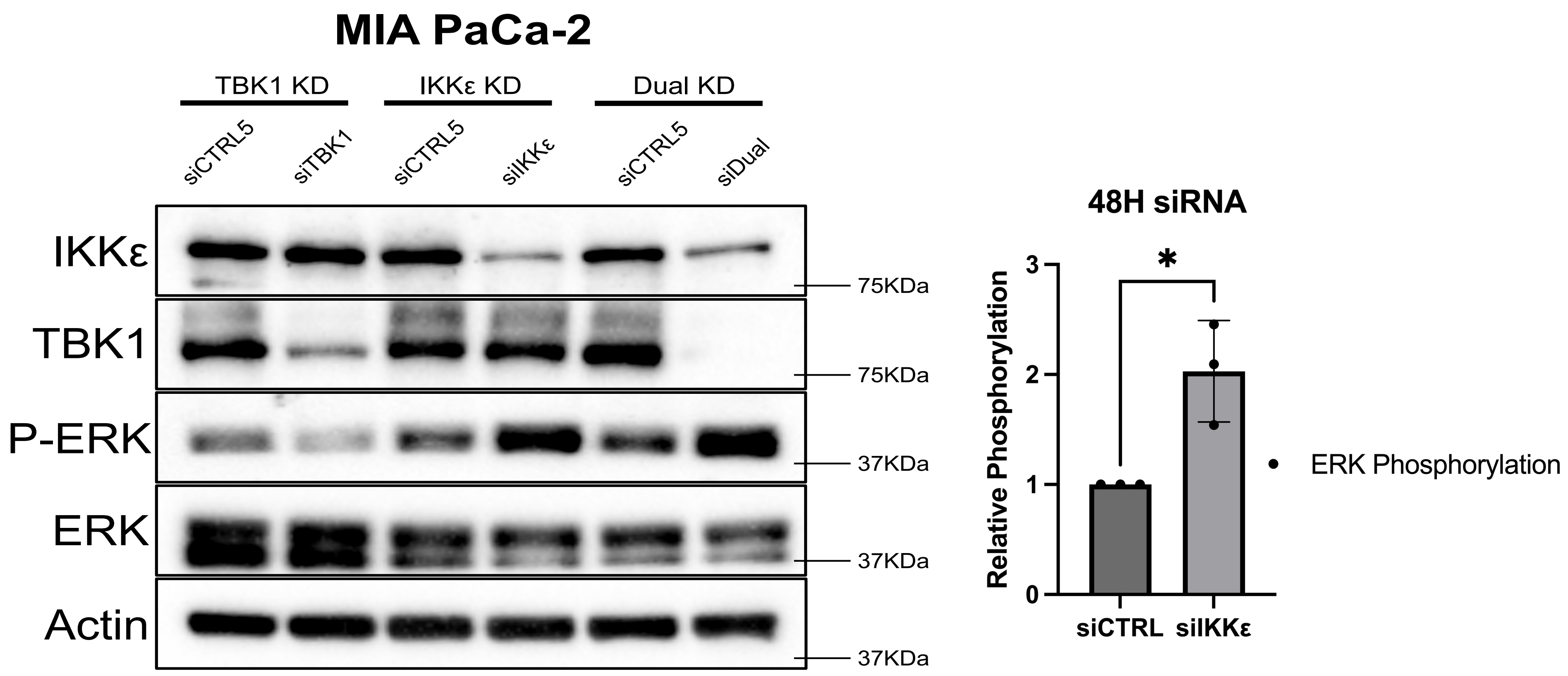

Supporting Information Figure 2 – IKKε Knockdown Increases ERK Phosphorylation in MIA PaCa-2 Cells

MIA PaCa-2 cells were treated for 48 hours with siRNA targeting TBK1, IKKε, or both (siDual) before harvesting and immunoblotting. Band intensity was determined using Fiji (ImageJ) and normalized to total ERK protein and Actin levels before an un-paired, parametric t-test was used to examine statistical significance (**P* = 0.0180). Blots are representative of three biological replicates and bar graph displays mean values with SD.

A)

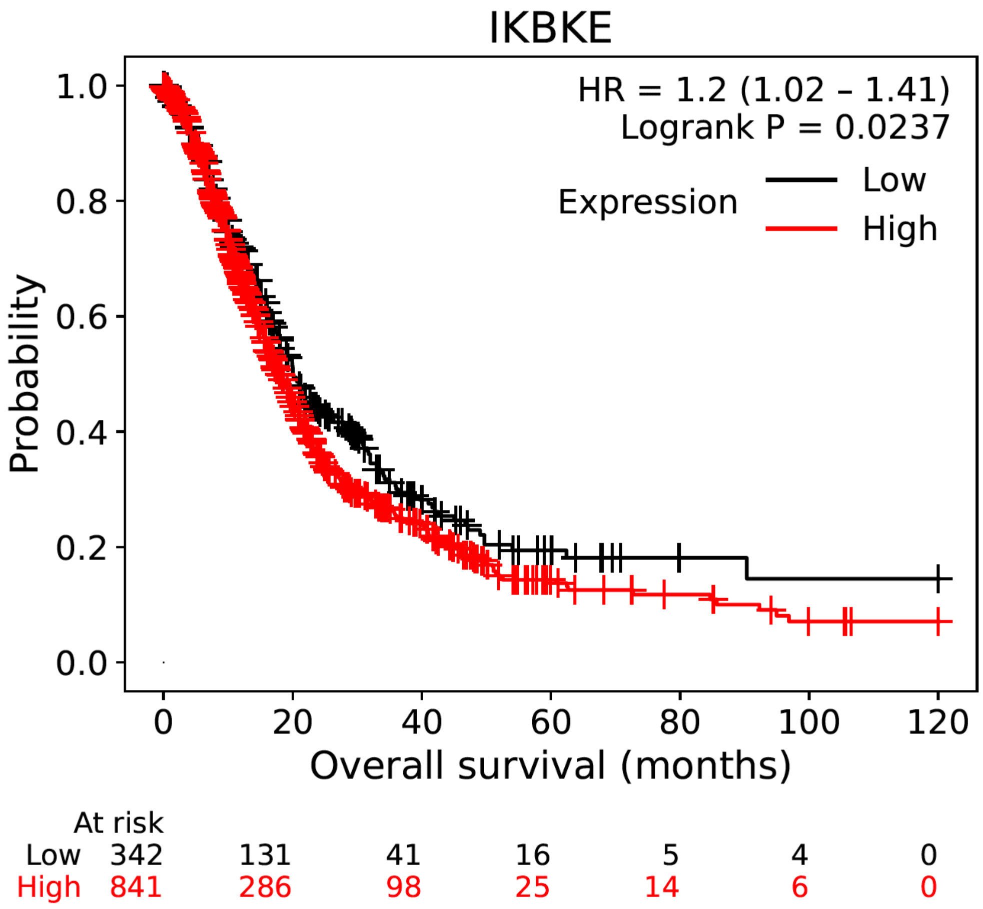

B)

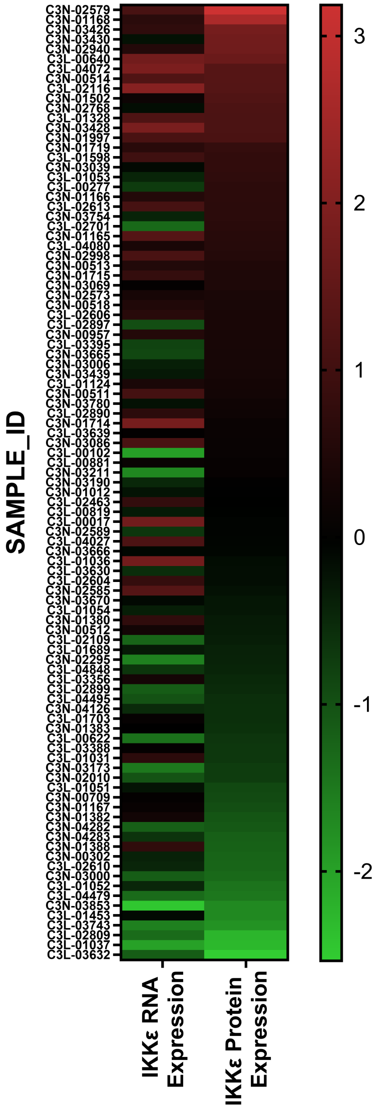

<u>Supporting Information Figure 3 – High IKKε Expression in PDAC Patients is Associated with Poorer Survival</u>

A) Relapse-free survival curves for PDAC patients were plotted for IKKε expression using the Kaplan-Meier Plotter website (n = 1183).

B) CPTAC-PDAC RNAseq data (n=140) were retrieved from cBioPortal. IKKε mRNA and protein abundance z-scores were plotted as a double gradient heat map.

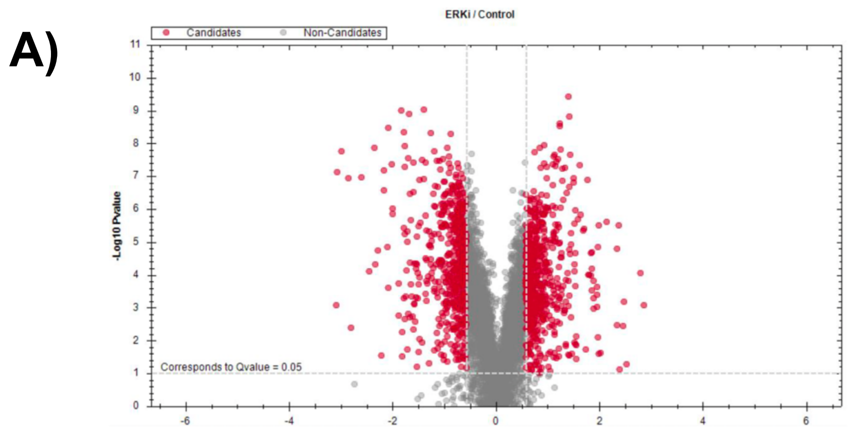

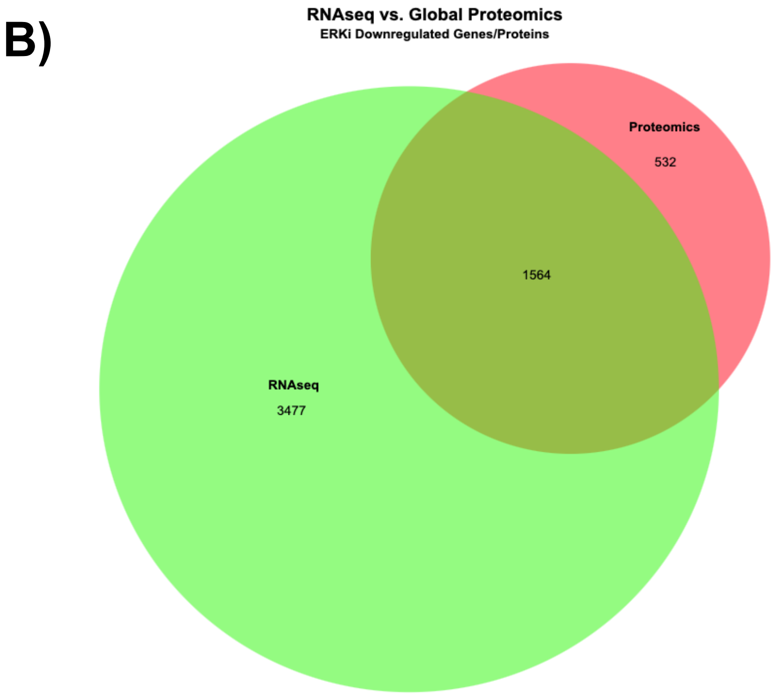

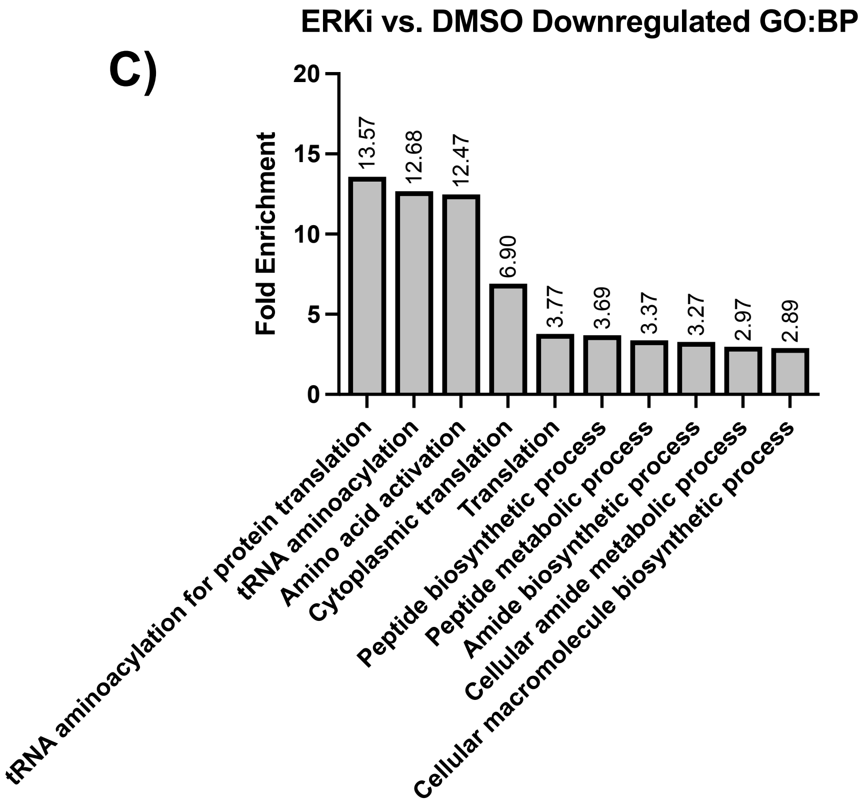

Supporting Information Figure 4 – Global Proteomic Profiling of ERKi-Treated MIA PaCa-2 Cells

A) MIA PaCa-2 cells were treated with SCH772984 for 24H before harvesting and global proteomic analysis (see methods). A volcano plot was generated by the UNC Proteomics Core (q-Value < 0.05, +/- 0.06 Fold Change).

B) Venn diagrams were created with BioVenn using proteomics data ($P < 0.05$) and RNAseq data ($P < 0.05$).

C) Gene ontology analysis was performed using biological pathways (GO:BP) downregulated in the protein-only results (532 proteins) when compared with the RNAseq samples.

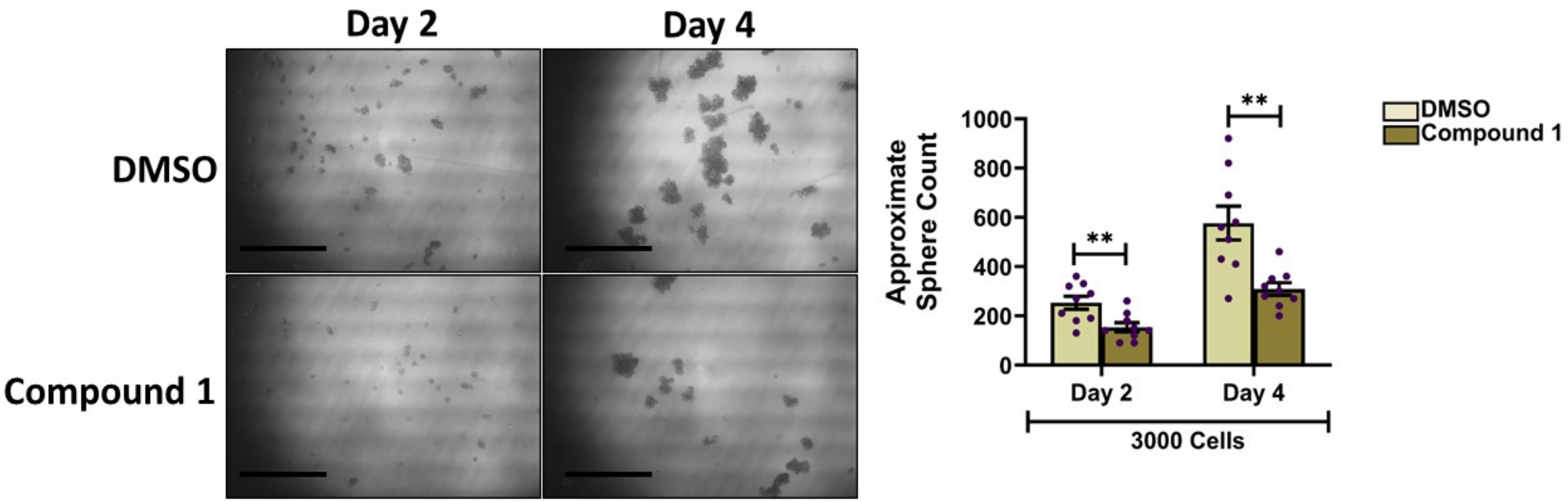

Supporting Information Figure 5 – TBK1/IKKε Inhibition Inhibits Tumor Sphere Formation of MIA PaCa-2 Cells

DMSO or Compound 1 treated MIA PaCa-2 cells were seeded for tumor sphere assays and images were taken on days 2, and 4. Quantification of tumor sphere formation was performed on days 2, and 4 (***P* = 0.006784, ***P* = 0.002090) (n = 3, and each replicate was seeded in 3 wells). Data was analyzed using unpaired multiple t-test. Scale bar: 100µm.
